# A systematic review of community Leg Clubs for patients with chronic leg ulcers

**DOI:** 10.1017/S1463423618000610

**Published:** 2018-08-30

**Authors:** Haya Abu Ghazaleh, Micol Artom, Jackie Sturt

**Affiliations:** 1 Research Associate, The Florence Nightingale Faculty of Nursing and Midwifery, King’s College London, London, UK; 2 Ph.D. Researcher, The Florence Nightingale Faculty of Nursing and Midwifery, King’s College London, London, UK; 3 Professor of Behavioural Medicine in Nursing, The Florence Nightingale Faculty of Nursing and Midwifery, King’s College London, London, UK

**Keywords:** Leg Club, leg ulcer, primary care, social care model, ulcer healing

## Abstract

**Aim:**

Appraise the evidence on the outcomes of Leg Clubs on ulcer healing, psychosocial outcomes, patient safety, cost and experiences of Leg Club members.

**Background:**

The Leg Club is a community-based social model of care in 30 UK locations and nine overseas for treating patients with chronic leg wounds. However, its cumulative effectiveness has not been reviewed to-date.

**Methods:**

Systematic review of primary research relating to the impact and quality of care of Leg Clubs treating patients with leg ulcers. Six electronic databases were systematically searched using the MeSH term ‘leg ulcer’, including other representative terms, in combination with ‘Leg Club’. The quality of individual studies was assessed using appraisal tools. The confidence in the quantitative evidence was evaluated using Grading of Recommendations Assessment, Development and Evaluation (GRADE); and the Confidence in the Evidence from Reviews of Qualitative Research (CERQual) assessed the quality of qualitative findings.

**Findings:**

A total of 17 relevant publications were identified. Out of the 17 articles, four publications represent findings from randomised controlled trial (RCT). Thus, evidence from 14 independent studies involving at least 532 participants were included in the synthesis of this review. The quality of the evidence varied across the different outcomes and were mostly low or of very low quality. Findings from one underpowered RCT from Australia reporting on clinical, patient-reported outcomes and economic outcomes were evaluated as moderate quality. Studies indicate that the Leg Club model has a positive impact on ulcer healing and recurrence, mood, sleep, quality of life and pain. Moreover, only three studies assessed wound infections and reported no infections had occurred during treatment at the Leg Clubs. Economic evaluations find Leg Clubs to be probably more cost-effective than usual care. Both patients and nurses projected positive views about the Leg Clubs, with particular emphasis on improved social interactions and delivery of patient-centred care.

## Introduction

A leg ulcer is defined as the loss of skin below the knee on the leg or foot, which takes more than two weeks to heal (National Institute for Health and Care Excellence, 2013). There are two main types of leg ulceration: venous and arterial. Venous leg ulceration is due to sustained venous hypertension, resulting from chronic venous insufficiency, whereas arterial leg ulceration is due to reduced arterial blood flow to the lower limb (Pannier and Rabe, 2013). In the UK population, the prevalence of leg ulcers is 0.56% (Guest *et al*., 2015). Venous leg ulcers are ~30 times more prevalent than arterial conditions (Guest *et al*., 2015). Leg ulcers are more frequent in women and incidences in the populace increase with age (Moffatt *et al*., 2004). Management of chronic wounds is estimated to cost the National Health Service (NHS) between £2.5 and £3.1 billion per annum, accounting for 3–4% of the healthcare budget (Posnett *et al*., 2009). Recent statistics reported that leg ulceration treatment costs the NHS £1.94 billion annually in 2012/2013, with higher incurred costs attributed to venous leg ulcers (£941 million) (Guest *et al*., 2017).

A systematic review of 23 studies illustrated that venous leg ulceration negatively impacts patients’ quality of life (QoL), impairs functioning and mobility and reduces social activities due to their symptoms (Green *et al*., 2014). Gold standard treatment for venous leg ulcer involves compression therapy to reduce venous hypertension (National Institute for Health and Care Excellence, 2013). Dressings are also required to prevent the bandage or compression hosiery from adhering to the wound (Royal College of Nursing, 2000; Scottish Intercollegiate Guidelines Network, 2010). A major problem among patients with both venous leg ulcers is the lack of compliance with their long-term treatment regimens (Jull *et al*., 2004; Raju *et al*., 2007) and compliance with leg ulcer treatment is acknowledged as an important determinant in leg ulcer healing and recurrence (Erickson *et al*., 1995). The weak rapport between patients and clinicians appeared to negatively influence patients’ adherence and concordance to treatment (Douglas, 2001). Limited knowledge, poor communication and increased nurses’ caseloads were some of the reasons that patients’ perceived to restrict their engagement with their carers (Douglas, 2001). Lack of compliance to long-term treatment regimens was reported to hinder healing and prompt ulcer recurrence in patients with venous and arterial leg ulcers (Erickson *et al*., 1995; Jull *et al*., 2004). Although there is a growing awareness of the problem of non-adherence to leg ulcer treatment, reasons for non-adherence are not fully understood (Van Hecke *et al*., 2011). Pain, treatment discomfort and poor lifestyle advice from practitioners were highlighted as key reasons for non-adherence to leg ulcer treatment according to patients (Van Hecke *et al*., 2009). Specifically for venous ulcers, beliefs that compression are unnecessary and uncomfortable had a significant detrimental effect on concordance. In contrast, beliefs that compression are worthwhile and prevented recurrence improved concordance (Van Hecke *et al*., 2009). Furthermore, compliance to treatment may vary according to treatment types, with studies showing that patient-reported compliance is higher in patients allocated to class three stockings compared with short-stretch compression bandages (Van Hecke *et al*., 2008). Defining effective ways to improve compliance to treatment for leg ulcers is therefore essential to enhance treatment outcomes (Van Hecke *et al*., 2008).

Publication of the UK National Institute for Health and Care Excellence (NICE) Clinical Guideline (CG) 168 on leg ulcers showed twofold increase in leg ulcer referrals (Davies *et al*., 2017). A Cochrane review of seven randomised controlled trials (RCTs) illustrated that compression use increases healing rates, and care delivery by specialist leg ulcer community clinics were superior to standard services offered by general practitioners (GP) and district nurses (Cullum *et al*., 2001). Hence, seeking specialised services in leg ulcer care is apparent to optimise patient clinical outcomes.

The Leg Club is a social model of care established to provide holistic treatment to people with lower-limb ulcerations. The Leg Club model offers treatment in an informal community setting by trained district or community nurses, allowing patients to socially engage with others during their visits and collectively receive treatment, sharing their experiences and offering peer support. Unlike primary care services, no appointments are required, allowing flexibility to access care and provides a fully integrated ‘well leg’ component in their treatment plan (Lindsay, 2004). There are currently 30 Leg Clubs operating in the United Kingdom, eight in Australia and one in Germany (The Lindsay Leg Club Foundation, 2018). Although new UK Leg Clubs have been established over the past 10 years, others have dissolved with growth hampered by insufficient evidence to inform clinical commissioning decisions. The clinical effects of a social model of wound care have not been well understood to-date. Given the high costs associated with delayed leg ulcer healing, evidence on the impacts and costs of the Leg Club model of care is warranted. This systematic review aims to identify published evidence on the impacts and quality of care of Leg Clubs on ulcer healing, psychosocial outcomes, patient safety and costs.

## Methods

### Search strategy

This systematic review was conducted in accordance to the Preferred Reporting Items for Systematic Reviews and Meta-analyses (PRISMA) guidance (Moher *et al*., 2009) (Appendix 1). The authors searched Medline, Embase, PsycINFO, Cinahl, Web of Science and the Cochrane Library. The last search was performed on 28 March 2017. The search utilised various terms to define ‘*leg ulcers*’ that was previously published in a Cochrane systematic review (Weller *et al*., 2016) in combination with ‘*leg club*’ to identify articles that addressed parameters pertaining to the Leg Club, including clinical responses, treatment safety, patient-reported outcomes, members’ experiences and economic impacts. The search strategy for Medline is shown in Appendix 2. Additional publications were identified through free-text searches on PubMed and Google Scholar, and reviewing the reference list of retrieved articles.

### Selection criteria

Quantitative and qualitative data examining ulcer healing, psychosocial outcomes, economic evaluations, treatment safety and/or experiences of members of the Leg Club were considered for inclusion. No restriction on year or study design was applied during the selection process and only English-written articles were included. Two researchers reviewed titles, abstracts or full-text publications to assess their eligibility and relevance.

### Data synthesis

No meta-analysis was performed due to heterogeneity of included studies and outcomes assessed. Hence, a narrative synthesis was conducted.

### Quality assessment

Included studies were evaluated for their methodological rigour and/or transparency in reporting their findings. Six tools were utilised to assess quality due to variances in study designs, including the Cochrane Collaboration tool for RCTs (Higgins *et al*., 2011), the Joanna Briggs Institute (JBI) critical appraisal checklist for qualitative studies (Lockwood *et al*., 2015), the JBI critical appraisal checklist for economic evaluation (Gomersall *et al*., 2015), the JBI critical appraisal checklist for case reports (Moola *et al*., 2017), the mixed-method appraisal tool (Pluye *et al*., 2011) or the quality assessment tool for observational cohort and cross-sectional studies devised by the National Institutes of Health (National Institutes of Health, 2014). No validated tools were available to evaluate audit reports. The overall quality of the study designs was rated as either good, fair or poor in accordance to each of the different assessment tool criteria. One author appraised the quality of each included study and another assessed 50% of the papers for accuracy. Discrepancies were resolved by consensus.

In addition, the Grading of Recommendations Assessment, Development and Evaluation (GRADE) approach was used to assess the certainty of the evidence for each quantitative outcome, evaluating risk of bias, inconsistency, indirectness, imprecision, publication bias, magnitude of effect, dose response and other plausible confounders (Ryan and Hill, 2016). Based on the GRADE system, the quality of the evidence was rated as high, moderate, low or very low. For qualitative studies, the Confidence in the Evidence for Reviews of Qualitative Research (CERQual) was applied assessing methodological limitations, coherence, adequacy and relevance (Lewin *et al*., 2018). The confidence of each review finding was judged as high, moderate, low or very low. Three review authors independently assessed the quality of the findings. Disagreements were resolved through consensus.

## Results

**Figure 1:**
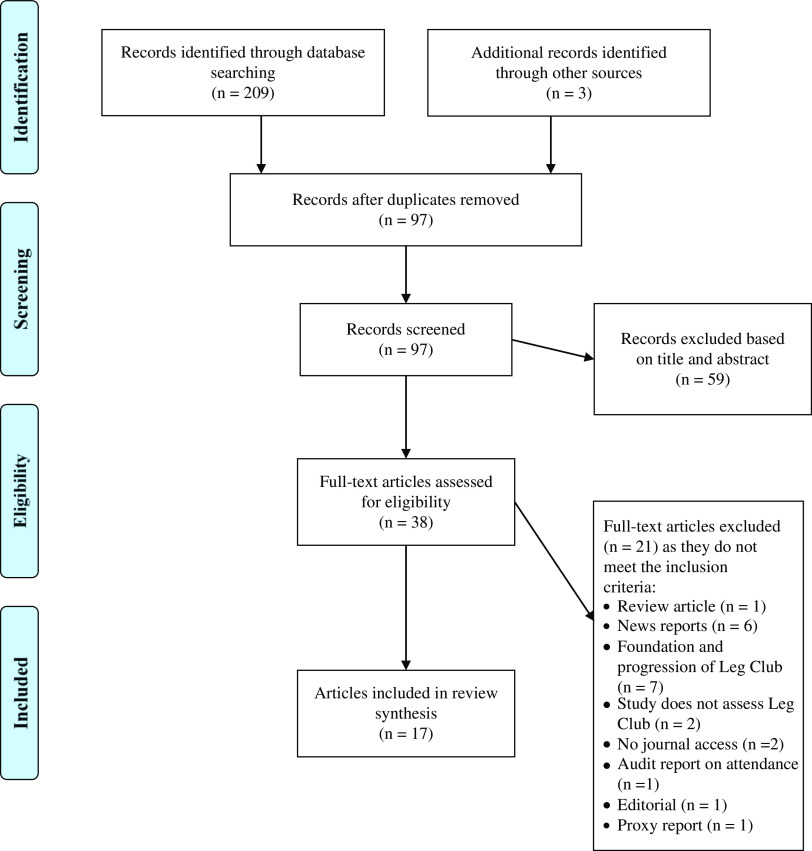
Literature search using the PRISMA paradigm.

The literature search retrieved 212 citations, whereby 115 articles were identified as duplicates and remaining 97 publications were screened for relevance based on information in their titles and abstracts. Full-text manuscripts were assessed for their eligibility and 17 papers were deemed relevant. Four out of the 17 publications represent data from the same study. Most studies excluded discussed either the history and foundation of the Leg Club (*n*=7), or provided news update reports on the progression and achievements of the Leg Club (*n*=6) (Figure 1).

### Overview of included studies

In this review, 17 publications representing 14 unique studies involving at least 532 participants were included, and the study characteristics are summarised in Table 1. In patients with leg ulcers, one RCT compared the effectiveness and assessed the health economics of standard care versus Leg Club (Edwards *et al*., 2005a; 2005b; 2009; Gordon *et al*., 2006), whereas most studies documented the experiences of Leg Club members using case reports (*n*=7) (Lindsay and Hawkins, 2003; Hampton and Lindsay, 2005; Shuter *et al*., 2011; Mew, 2015; Renyi and Hampton, 2015; Hampton, 2016; Wright, 2016), mixed-method approaches (*n*=2) (Elster *et al*., 2013; Upton *et al*., 2015), cross-sectional studies (*n*=2) (Lindsay, 2004; Clark, 2012) or a qualitative method (Stephen-Haynes, 2010). Moreover, two papers reported audit data on leg ulcer recurrence and healing rates, cost-savings and compliance to treatment (Lindsay, 2001; Elster *et al*., 2013); 11 studies were conducted in the United Kingdom and three in Australia.

**Table 1:** Summary of included articles

Author (year)	Country, population, design	Sample size	Study aims	Outcome(s)
RCT				
Study 1				
Edwards *et al*. (2005a)	Country: Australia Population: patients with venous leg ulcers	33	Evaluate effectiveness of Leg Club versus nurse home care in managing leg ulcer care. FU: 12 weeks	Healing rates, QoL, health status, functional ability, pain levels
Edwards *et al*. (2005b)	Country: Australia Population: patients with chronic venous leg ulcers	56	Determine effectiveness of Leg Club versus home visit by community nurse in managing leg ulcer care. FU: 12 weeks	Healing rates, pain levels, mood, sleep, functional ability
Gordon *et al*. (2006)	Country: Australia Population: patients with venous leg ulcers	56	Evaluate cost-effectiveness of Leg Club versus standard care (home visit)	Total cost to service provider, community and patients; cost per healed ulcer to service provider, community and patients; cost per reduced pain score to service provider, community and patients
Edwards *et al*. (2009)	Country: Australia Population: patients with venous leg ulcers	67	Investigate effectiveness of Leg Club *vs.* nurse home care visit. FU: 12 and 24 weeks	QoL, morale, mood, pain level, self-esteem, social support, physical functioning, healing
Qualitative				
Study 2				
Stephen-Haynes (2010)	Country: UK Population: Leg Club members	100	Evaluate the perception of Leg Club members and nurses on the model of care delivery	Five recurrent themes or keywords to describe model of care
Mixed method				
Study 3				
Elster *et al*. (2013)	Country: UK Population: patients with leg ulcers Design: pilot audit data+questionnaire+informal interview	ND	Explore effectiveness (six months) and experiences of Leg Club members	Healing and leg ulcer recurrence rates, member experience
Study 4				
Upton *et al*. (2015)	Country: UK Population: Leg Club members Design: survey+interview, seven centres	49	Explore the potential association between social support and well-being	Satisfaction, self-esteem, mood, social situation, healing, well-being, wound healing and duration
Descriptive/cross-sectional				
Study 5				
Clark (2012)	Country: UK Population: Leg Club members Design: questionnaire, seven centres	124	Assess experience of Leg Club attendees	Acceptability and satisfaction levels, social experience, care delivery
Study 6				
Lindsay (2004)	Country: UK Population: Leg Club members Design: survey, two centres	ND	Explore committee member’s views on Leg Clubs	Ambiance, social support, uptake of knowledge
Case report				
Study 7				
Hampton and Lindsay (2005)	Country: UK Population: T1D with bilateral varicose eczema and leg ulcers	1	Document clinical history and experience of patient attending the Leg Club	Healing, care delivery, concordance to treatment, social support
Study 8				
Hampton (2016)	Country: UK Population: patient with leg ulcer	1	Evaluate the experience of patient attending the Leg Club	Healing, mood, satisfaction
Study 9				
Lindsay and Hawkins (2003)	Country: UK Population: patients with venous leg ulcers	1	Document clinical history and experience of attending the Leg Club	QoL, healing, self-esteem
Study 10				
Mew (2015)	Country: UK Population: patients with leg ulcers	2	Monitor medical history and experience of attending the Leg Club	Social support, healing, QoL
Study 11				
Renyi and Hampton (2015)	Country: Australia Population: diabetic with leg ulcer and lymphoedema	1	Monitor medical history and experience of attending the Leg Club	Social support, healing, QoL
Study 12				
Shuter *et al*. (2011)	Country: Australia Population: Leg Club members	3	Examine the psychosocial benefits and outcomes for patients engaged in Leg Clubs	Social changes, self-esteem, social support and quality of care
Study 13				
Wright (2016)	Country: UK Population: patients with venous leg ulcers	1	Assess medical history and experience at the Leg Club	Healing, self-esteem, mood, social support
Study 14				
Lindsay (2001)	Country: UK Population: Leg Club members Design: two centres	93	Assess the clinical and economic benefits of Leg clubs (11 months)	Cost savings, treatment compliance, healing and infection rates

RCT=randomised controlled trial; FU=follow-up; QoL=quality of life; ND=not determined; T1D=type 1 diabetes.

### Quality appraisal of individual studies

The quality of included articles was evaluated using multiple tools due to heterogeneity of study designs. Information from RCTs and the health economics publication were deemed good quality with low risk of bias across most of the domains, including randomisation, reporting of outcomes and attrition. However, details on allocation concealment and blinding of assessors were ambiguous and blinding of participants was not possible. The qualitative study fulfilled all the assessment criteria, illustrating low risk of bias. Both cross-sectional studies were evaluated as poor quality due to inadequacy in methodological description, data analyses and lack of information on blinding of outcome assessors. One of the mixed-method studies was deemed fair and the other was evaluated as poor in quality, lacking information on sampling, attrition and study limitations. Moreover, both studies failed to address research bias in their methodology, affecting the credibility of their results. The results showed two out of eight case studies were judged as good quality, whereas four were evaluated as fair and one case report was appraised as poor quality. Case reports graded as fair or poor provided inadequate information on diagnostic tests and assessment methods. Nonetheless, all case studies provided clear details on medical history and clinical conditions of their participants. Given that this is an exploratory area of research, all relevant studies and audit reports, irrespective of study quality, were included and study limitations and potential sources of bias are highlighted in the discussion section.

### Clinical impact of the Leg Club

Seven separate studies with a minimum of 209 participants assessed the clinical outcomes of leg ulcer patients attending the Leg Club, illustrating that treatment at the Leg Club may improve healing rates and may reduce ulcer recurrence (Lindsay and Hawkins, 2003; Edwards *et al*., 2005a; 2005b; 2009; Hampton and Lindsay, 2005; Elster *et al*., 2013; Upton *et al*., 2015; Hampton, 2016; Wright, 2016). A pilot and feasibility RCT conducted by the same group in Australia compared the effectiveness of the Leg Club to standard care demonstrating that the Leg Club model may be superior to home nursing visits with significant improvement in ulcer healing within 12 weeks, with 69–77% reduction in mean ulcer area versus 10–11% reduction, respectively (Edwards *et al*., 2005a; 2005b). Ulcer area was further significantly reduced at 24 weeks in both treatment groups (Edwards *et al*., 2009). Although, the healing rate was quicker in the intervention arm (0.267 cm^2^/week) than the control group (0.089 cm^2^/week) (Edwards *et al*., 2009). Ulcer healing at six months differed between Leg Club locations, whereby improvements were greater in Leg Clubs in Australia (60%) (Edwards *et al*., 2009) compared with United Kingdom (42%) (Elster *et al*., 2013). The overall quality of the evidence from the two RCTs is high, illustrating that the ulcer care at the Leg Clubs was associated with better health outcomes than usual care.

In one study, venous eczema and oedema were less prevalent in patients receiving treatment at the Leg Club than those offered standard care (Edwards *et al*., 2005a), and an audit report from the Barnstaple Leg Club demonstrated that leg ulcer recurrence was low (Elster *et al*., 2013). Other variables evaluating ulcer care were gathered from testimonials from Leg Club members claiming that their ulcer wounds had improved or healed within 11–24 weeks (Lindsay and Hawkins, 2003; Hampton and Lindsay, 2005; Elster *et al*., 2013; Renyi and Hampton, 2015; Hampton, 2016; Wright, 2016). The small number of events in the analysis and the lack of a control group or comparator in these studies affect the credibility of the results, deeming the data insufficient to verify the impact of care from the Leg Club on ulcer recurrence.

### Patient safety of Leg Club members

One RCT presented data on the incidence of a wound infection at 12 weeks in one leg ulcer patient receiving standard care; whilst one patient in the Leg Club intervention arm developed a new ulcer (Edwards *et al*., 2005a). Audit data recorded over 11 months from two Leg Clubs in the United Kingdom (*n*=93) reported that none of their members presented any clinical infections (Lindsay, 2001). This was similarly noted in one case who attended a Leg Club in Australia for three years (Renyi and Hampton, 2015). No other publications reported wound infections in their findings. Although the results are consistent across the three studies, the overall quality of the evidence from the RCT is low and very low for the non-RCTs, which is mainly due to the small number of participants investigated (*n*=127), increasing the probability of imprecision in the findings.

### Patient-reported outcomes from Leg Club interventions

Evidence from four separate studies demonstrated that the Leg Club may enhance QoL, functional abilities, morale, mood and self-esteem (Lindsay and Hawkins, 2003; Edwards *et al*., 2005b; 2009; Shuter *et al*., 2011; Upton *et al*., 2015). Decreased levels of pain were experienced by Leg Club patients, which may be directly associated with improved sleep, mood and normal working habits (Edwards *et al*., 2005b). Social support and depression scores did not differ between the two arms at 24 weeks (Edwards *et al*., 2009). One questionnaire-based study reported that the Leg Club model may enhance patient understanding of their leg condition, which may help better manage their ulcer care (Clark, 2012). Two studies illustrated that patients were compliant to treatment offered by the Leg Club (Lindsay, 2001; Hampton and Lindsay, 2005). However, no comparative control group was included in their study designs to determine whether different treatment modalities for leg ulcers influence patient compliance. Overall, the evidence is of low certainty due to the small number of total participants in the analysis.

In one study of 49 participants, more than 50% of members mentioned that attendance at the Leg Club improved their social situation and enhanced their well-being, which may have positively influenced treatment success (Upton *et al*., 2015). However, given that the evidence is very low quality, it is not certain whether there is a direct association between the social aspect of the Leg Club and ulcer healing.

### Experience and perception of the Leg Club model: patients’ perspective

A total of 12 studies with at least 283 participants, mostly case reports (*n*=7), documented the experiences of Leg Club members assessing the impact of care delivery and model concept as a whole. Ten of these explored the views of UK patients (Lindsay and Hawkins, 2003; Lindsay, 2004; Hampton and Lindsay, 2005; Stephen-Haynes, 2010; Shuter *et al*., 2011; Clark, 2012; Elster *et al*., 2013; Mew, 2015; Renyi and Hampton, 2015; Upton *et al*., 2015; Hampton, 2016; Wright, 2016). Regardless of location, leg ulcer patients expressed positive views about the Leg Club, emphasising the advantage of peer support during the healing process (Hampton and Lindsay, 2005; Clark, 2012; Elster *et al*., 2013; Mew, 2015; Renyi and Hampton, 2015; Hampton, 2016; Wright, 2016). When compared with NHS facilities, more than 50% of members reported to attend the Leg Club because they enjoyed the social atmosphere and had more confidence in the advice and/or treatment received (Clark, 2012). Moreover, 71 out of 86 current members rated that they were very satisfied with services received at the Leg Club (Clark, 2012). Results from an interpretive phenomenological analyses of Leg Club attendees highlighted the importance of social interaction as well as the accessibility and continuity of care received at the Leg Club (Upton *et al*., 2015). These views were similarly echoed by participants from an explorative qualitative study with great emphasis on sociability and quality of care (Stephen-Haynes, 2010). The Leg Club was described as friendly and welcoming, and attending patients appreciated the care and quality of services (Lindsay, 2004; Stephen-Haynes, 2010; Shuter *et al*., 2011; Clark, 2012; Elster *et al*., 2013). Based on the CERQual assessment, the evidence on social interaction was considered moderate due to serious concerns regarding adequacy of data, whereas the confidence in the finding about the high quality of care in the Leg Clubs was considered low due to limited thin data from three studies, comprising qualitative components in their research, with moderate methodological limitations. Findings from five publications reported that patients regained their sense of purpose and had better control over their own lives, with greater ownership in their treatment plan (Lindsay and Hawkins, 2003; Lindsay, 2004; Shuter *et al*., 2011; Clark, 2012; Wright, 2016). Given that most of the evidence is derived from non-RCTs with a small number of participants, the overall quality of the observational studies is considered very low according to the GRADE framework.

### Experience and perception of the Leg Club model: nurses’ perspective

There is very low quality evidence from one qualitative study exploring the views of healthcare providers (*n*=15) from two different Leg Clubs in the United Kingdom (Stephen-Haynes, 2010). Overall, they described their jobs as ‘*challenging*’ and feeling ‘*tired*’. Nonetheless, they described the Leg Club as a hospitable environment for staff and clients. Common emerged themes derived from staff members included ‘*education*’, ‘*camaraderie*’ and ‘*empowerment*’, signifying a collaborative learning environment allowing both patients and staff to grow. Most importantly, nurses felt patients were empowered to take ownership in their treatment. The confidence in the evidence on the nurses’ perception was deemed moderate due to serious concerns of limited data derived from only one qualitative study.

### Economic impact of the Leg Club

The moderate quality evidence from one RCT in Australia involving 67 participants demonstrated that the Leg Club probably incurs lower costs than home nurse visits by $1727 (approximately £1385) during a three-month period (Gordon *et al*., 2006). Moreover, medical supply expenses were valued at 30% less for Leg Club compared with home nursing (Gordon *et al*., 2006). Although total expenditure to the community for Leg Club was 20% higher than home nursing, the Leg Club leads to higher healing rates than standard care and had lower costs per healed ulcer during three ($1019 versus $1571) and six months ($1546 versus $2061). Overall, the Leg Club model appears to probably offer cost advantages over usual home nursing care (Gordon *et al*., 2006). Only one UK audit report examining the total cost of wound management in patients attending the Leg Club during 11 months, and 73% of the population incurred £50, whereas 13% spent more than £200 to treat their condition (Lindsay, 2001).

## Discussion

### Quality of the evidence

This review considered the evidence from a wide range of study designs developed in two countries supporting the Leg Club model of care. The quality of findings ranged from moderate to very low across the different quantitative outcomes, and the confidence in qualitative findings was moderate to low primarily due to concerns in methodological design and data adequacy as assessed by CERQual. The main limiting factor that downgraded quality in most quantitative outcomes in accordance to the GRADE framework was the imprecision of results due to the small number of participants included in the analysis. Moreover, the lack of a comparator to evaluate the effectiveness of the Leg Club interventions is another limitation associated with most studies. Although, it can be argued that the evidence for clinical outcomes and patient-reported outcomes from the one RCT could be evaluated as moderate quality since a large magnitude effect and dose-response effect were illustrated. However, blinding of participants and outcome assessors were not possible in this RCT, and as such increases the probability of performance bias and detection bias. Moreover, inclusion of data from non-RCT studies exposes further risk of bias affecting the credibility of the overall results. Therefore, the evidence was assessed as low or very low quality for most outcomes.

Although the evidence is rich in diversity of outcomes, it is weak in providing robust scientific direction for healthcare commissioners who may wish to explore this model in community settings. The higher quality evidence showing improvements in ulcer healing and other patient-reported outcomes was from one underpowered RCT conducted in Australia. No experimental evidence exists for the majority of Leg Clubs operating in the United Kingdom. Australian findings may not be mirrored in the United Kingdom, such that healing rates in the United Kingdom were nearly two-thirds of that achieved in the Australian context (Edwards *et al*., 2009; Elster *et al*., 2013). However, no demographic information about the study population was described by Elster *et al*. (2013), and as such it is not possible to compare patient characteristics from the two studies or draw any inferences on the factors that may influence the reported differences in healing rates between the Leg Clubs in the two countries. Whilst nurses reported positive working experiences and patient outcomes in the United Kingdom, differences between healthcare contexts in Australia and the United Kingdom require further investigation. Clinical practices, context and capacity of Leg Club location, nurse training and availability may influence the varied outcomes between the two countries.

Two studies found that treatment concordance improved during attendance at the Leg Club (Lindsay, 2001; Hampton and Lindsay, 2005). However, no correlative assessments were conducted to determine whether Leg Club attendance was positively associated with treatment concordance and what the mechanisms of increased concordance might be. NICE guidance recommends specialist community wound clinics over home-based GP/community nurse-led treatment (National Institute for Health and Care Excellence, 2013). Future evaluations of the Leg Club in the United Kingdom need to be undertaken in this context where the comparator is one of current best practice such as a specialist community clinic. Treatment concordance has been found to improve in specialist multidisciplinary clinics compared to usual NHS care in other conditions (Van Groenendael *et al*., 2015). The question of cost-effectiveness may be crucial in determining best practice for the management of chronic leg wounds in the community.

### Strengths and limitations

Although our review identified relevant articles systematically, it was difficult to draw conclusive inferences due to variability in study designs and assessment tools. There were numerous limitations related to the studies, including minimal detail of sampling strategies, allocation concealment, blinding of participants and assessors, small sample sizes, and lack of clarity on study and analytic methods. Therefore, it was difficult to evaluate the validity and rigour of the findings. Furthermore, due to the nature of the methodology of surveys, audits and case reports, no comparators were included; thus rendering their data as inconclusive. In addition, the discrepancy in the different sample sizes reported from the same RCT could not be resolved despite attempts in contacting the authors. Consequently, we believe that the evidence published at different time points represent findings from preparatory work that includes single feasibility (Edwards *et al*., 2005a) and pilot (Edwards *et al*., 2005b) testing stages, as well as data from the later full RCT (Edwards *et al*., 2009).

### Implications for research

This review highlights the potential of the Leg Club social model of care to make contributions to reducing the burden for people with chronic leg wounds and the costs associated with these conditions. A few Leg Clubs are starting to be commissioned by NHS local commissioning groups who believe that the cost-savings and improvement in care demonstrated by local audits are convincing (Lindsay, 2001; Elster *et al*., 2013). NICE guidance relies on high-quality evidence, which is required of Leg Club care to enable commissioners and health professionals to make sound scientific and cost-effectiveness decisions on referral of patients presented with leg ulcers. The overall quality of the evidence is lacking and warrants future research using more robust RCT designs to determine the efficacy of Leg Club interventions on ulcer healing.

## Conclusion

The Leg Club holds potential for providing cost-effective specialist community-based wound care. A fully powered UK RCT is needed with appropriate comparator groups to ascertain the value and contribution of Leg Clubs to the UK NHS.

## Appendix 1: PRISMA checklist

**Table:**

Section/topic	Item no.	Checklist item	Included (Yes/No/NA)	Item location (section)
Title				
Identification	1a	Identify the report as a protocol of a systematic review	Yes	Title, Abstract, Introduction, Method
Update	1b	If the protocol is for an update of a previous systematic review, identify as such	NA	
Registration	2	If registered, provide the name of the registry and registration number	NA	
Authors				
Contact	3a	Provide name, institutional affiliation and email address of all protocol authors; provide physical mailing address of corresponding author	Yes	Cover page
Contributions	3b	Describe contributions of protocol authors and identify the guarantor of the review	Yes	Cover page, Author contribution
Amendments	4	If the protocol represents an amendment of a previously completed or published protocol, identify as such and list changes, otherwise, state plan for documenting important protocol amendments	NA	
Support				
Sources	5a	Indicate sources of financial or other support for the review	Yes	Funding, Acknowledgements
Sponsor	5b	Provide name for the review funder and/or sponsor	NA	
Role of sponsor/funder	5c	Describe roles of funder(s), sponsor(s), and/or institution(s), if any, in developing the protocol	Yes	Acknowledgements
Introduction				
Rationale	6	Describe the rationale for the review in the context of what is already known	Yes	Introduction
Objectives	7	Provide an explicit statement of the question(s) the review will address with reference to participants, interventions, comparators and outcomes (PICO)	Yes	Introduction
Methods				
Eligibility criteria	8	Specify the study characteristics to be used as criteria for eligibility for the review	Yes	Method
Information sources	9	Describe all intended information sources with planned dates of coverage	Yes	Method
Search strategy	10	Present draft of search strategy to be used for at least one electronic database, including planned limits, such that it could be repeated	Yes	Appendix 2
Study records				
Data management	11a	Describe the mechanism(s) that will be used to manage records and data throughout the review	Yes	Method
Selection process	11b	State the process that will be used for selecting studies through each phase of the review	Yes	Method
Data collection process	11c	Describe planned method of extracting data from reports, any processes for obtaining and confirming data from investigators	Yes	Method
Data items	12	List and define all variables for which data will be sought, any pre-planned data assumptions and simplifications	Yes	Abstract, Method
Outcomes and prioritisation	13	List and define all outcomes for which data will be sought, including prioritisation of main and additional outcomes, with rationale	Yes	Introduction
Risk of bias in individual studies	14	Describe anticipated methods for assessing risk of bias of individual studies, including whether this will be done at the outcome or study level, or both; state how this information will be used in data synthesis	Yes	Method
Data				
Synthesis	15a	Describe criteria under which study data will be quantitatively synthesised	NA	
	15b	If data are appropriate for quantitative synthesis, describe planned summary measures, methods of handling data, and methods of combining data from studies, including any planned exploration of consistency	NA	
	15c	Describe any proposed additional analyses	NA	
	15d	If quantitative synthesis is not appropriate, describe the type of summary planned	Yes	Results
Meta-bias(es)	16	Specify any planned assessment of meta-bias(es)	Yes	Abstract, Methods
Confidence in cumulative evidence	17	Describe how the strength of the body of evidence will be assessed (eg, GRADE)	Yes	Abstract, Methods, Results, Discussion

## Appendix 2: Search strategy in Medline

1. Exp Leg Ulcer/
2. varicose ulcer$.mp.
3. venous ulcer$.mp.
4. leg ulcer$.mp.
5. foot ulcer$.mp.
6. (feet adj ulcer$).mp.
7. stasis ulcer$.mp.
8. (lower extremit$ adj ulcer$).mp.
9. crural ulcer$.mp.
10. ulcus cruris.mp.
11. 1 or 2 or 3 or 4 or 5 or 6 or 6 or 7 or 8 or 8 or 9 or 10
12. 12. ‘Leg Club’.mp.
13. 11. and 12.
